# Incidence and predictors of mortality among neonates with respiratory distress syndrome admitted to neonatal intensive care units in Ethiopia: a systematic review and meta-analysis of evidence from two regional states

**DOI:** 10.1186/s12887-026-06877-5

**Published:** 2026-04-17

**Authors:** Daniel Mulat Eshetu, Thomas Kidanemariam Yewodiaw, Ambachew Motbaynor Wubaye, Birhanu Feleke Shitu, Amare Mebrat Delie, Aysheshim Belaineh Haimanot, Yezbie Wagaw Ayicheh, Adugna Mengesha Mulugeta, Anley Shiferaw Enawgaw, Yihenew Getahun Ambaw, Mequanent Dessie Bitewa, Smegnew Gichew Wondie, Tadele Derbew Kassie, Zinie Abita Mengie, Simegnew Adugna Kallu, Berhanu Ayana Tafere, Tesfaye Shumet Mekonnen, Mikias Getahun Molla

**Affiliations:** 1 Department of Public Health, College of Medicine and Health Sciences, Injibara University, Injibara, Ethiopia; 2 Amhara Region Emergency Operation Center, Medical Officer at International Medical Corps, Gondar Field Office, Gondar, Ethiopia; 3 Department of Veterinary Science, College of Agriculture and Environmental Sciences, Debre Tabor University, Debre Tabor, Ethiopia; 4 Northern Resilience Cluster Office, Jhpiego, Ethiopia; 5 Department of Public Health, College of Medicine and Health Sciences, Debre Markos University, Debre Markos, Ethiopia; 6 Department of Veterinary Medicine, College of Agricultural Sciences, Woldia University, Woldia, Ethiopia; 7 Department of Public Health, College of Medicine and Health Sciences, Mizan-Tepi University, Mizan Aman, Ethiopia; 8 College of Veterinary Medicine, Haramaya University, Dire Dawa, Ethiopia; 9 Department of Midwifery, College of Medicine and Health Sciences, Debre Markos University, Debre Markos, Ethiopia

**Keywords:** Incidence, Neonatal Mortality, Respiratory Distress Syndrome, Predictors, Ethiopia

## Abstract

**Background:**

Neonatal respiratory distress syndrome (RDS) is a leading cause of neonatal morbidity and mortality, particularly in low-resource settings. In Ethiopia, evidence on the incidence of mortality and its predictors among neonates with RDS is limited and fragmented. Therefore, this systematic review and meta-analysis, based on data from two regional states, aimed to answer the following question: *“What is the pooled incidence of mortality*,* and what are the key predictors of death among neonates with RDS in Ethiopia?”*

**Methods:**

International databases, PubMed, HINARI, Google Scholar, Embase, Scopus, and Ethiopian university repositories were searched for studies published between January 1, 2000, and February 10, 2026. Studies reporting the incidence of mortality and/or predictors among neonates with RDS in Ethiopia were eligible. Both published and gray literature were included. Study quality was assessed using the Joanna Briggs Institute critical appraisal checklist. The pooled incidence of mortality was estimated using the DerSimonian–Laird random-effects model. Heterogeneity was assessed with I² statistics and Cochran’s Q test, and publication bias was evaluated using funnel plots and Egger’s test. Associations with mortality were summarized using pooled hazard ratios. Subgroup analyses and sensitivity analysis explored sources of variation.

**Results:**

From 1,982 records, five retrospective cohort studies, including 2,121 neonates and 12,272 neonatal-days of follow-up, were included. The pooled incidence of mortality was 62 deaths per 1000 neonatal-days (95% CI: 56–69; I² = 51.25%), corresponding to a case fatality rate of 36% (95% CI: 32.5–40.1%) within 28 days of birth. Mortality predictors were a birth weight of 1500–2499 g (HR 1.85; 95% CI: 1.41–2.43), very low birth weight (HR = 4.24; 95% CI: 2.30–7.83), preterm birth (HR = 1.77; 95% CI: 1.27–2.48), perinatal asphyxia (HR = 2.11; 95% CI: 1.31–3.41), and multiple pregnancies (HR = 2.25; 95% CI: 1.63–3.10), whereas maternal antenatal corticosteroids reduced mortality (HR = 0.33; 95% CI: 0.09–0.56). Sensitivity analyses indicated robust results; no substantial publication bias was detected.

**Conclusions:**

Neonatal mortality due to respiratory distress syndrome in Ethiopia, based on evidence from two regional states (Amhara and Oromia), remains high. Preterm, asphyxiated, and low-birth-weight neonates are at increased risk of death, while antenatal corticosteroid use is protective. These findings highlight the importance of strengthening the timely administration of antenatal corticosteroids and improving the clinical management of high-risk neonates to reduce mortality and improve survival outcomes in low-resource settings. However, as the analysis is based on a limited number of studies from only two regions, the results should be interpreted with caution.

**PROSPERO registration number:**

CRD420261291462

**Supplementary Information:**

The online version contains supplementary material available at 10.1186/s12887-026-06877-5.

**Table:** 

Text box 1. Contributions to the literature
• Provides the first systematic synthesis of neonatal mortality due to respiratory distress syndrome in Ethiopia based on evidence from available literatures from two reginal states.
• Highlights key clinical and demographic risk factors relevant to low-resource settings.
• Demonstrates the protective impact of maternal antenatal corticosteroid use on newborn survival.
• Contextualizes respiratory distress syndrome-related mortality within broader neonatal health challenges in low- and middle-income countries.
• Supplies evidence to guide public health policies, improve neonatal care practices, and prioritize interventions to reduce preventable neonatal deaths.

## Background

Neonatal respiratory distress syndrome (RDS), also known as hyaline membrane disease, is one of the most common causes of respiratory distress in newborns immediately after birth. It is a devastating inflammatory lung condition characterized by diffuse alveolar damage, increased alveolar-capillary membrane permeability, and impaired gas exchange, often leading to respiratory failure [1–3]. RDS is a major reason for admission to neonatal intensive care units (NICUs) worldwide, and it remains a leading contributor to neonatal morbidity and mortality [4]. Clinically, RDS can be identified by abnormal respiratory rates, tachypnea (> 60 breaths/min), bradypnea (< 30 breaths/min), or apnea, along with signs of labored breathing, such as expiratory grunting, nasal flaring, chest wall retractions, and, in severe cases, cyanosis [5]. Respiratory morbidity affects approximately 15% of term infants and 29% of late preterm infants in critical care settings, with significantly higher prevalence among neonates born before 34 weeks of gestation [6].

The clinical course of RDS is often severe, with rapid progression from respiratory distress to life-threatening complications if not promptly recognized and managed. Up to 75% of neonatal deaths within the first week of life are linked to respiratory distress conditions, emphasizing the critical need for early intervention [6, 7]. Neonates with respiratory distress are two to four times more likely to die than those without, highlighting the importance of timely prevention and effective management [8]. Globally, the incidence of RDS varies, affecting 7–8% of live births, with higher rates among preterm (≈ 30%) and post-term (≈ 20%) infants, and lower rates in term infants (≈ 4%). RDS contributes to roughly 20% of all neonatal deaths [9]. The burden is disproportionately higher in low-resource regions, with the highest neonatal mortality rates observed in South Asia and sub-Saharan Africa [6]. In Ethiopia, case fatality among neonates with RDS has been reported at around 32% [10].

Several factors increase the risk of mortality from RDS, including prematurity, low birth weight, neonatal sepsis, and maternal or pregnancy-related complications such as gestational diabetes, premature rupture of membranes, pre-eclampsia, perinatal infection, and abnormal placental implantation or detachment [2]. Standard management of RDS involves surfactant replacement therapy, respiratory support through ventilators or continuous positive airway pressure (CPAP), oxygen supplementation, antibiotics to prevent or treat infection, and supportive care including thermal regulation [11].

Despite some progress in reducing neonatal mortality, Ethiopia continues to face significant challenges. The neonatal mortality rate decreased from 33 per 1000 live births in 2019 to 27 per 1000 in 2020 [2], and further to 25 per 1000 in 2024/25 [12]. While this trend represents improvement, it remains far from the Sustainable Development Goal target of 12 per 1000 live births by 2030 [13]. Previous studies in Ethiopia on RDS mortality have been fragmented and limited in scope, lacking national representativeness. To address this gap, this systematic review and meta-analysis aimed to determine the pooled incidence and predictors of mortality among neonates with respiratory distress syndrome in Ethiopia, providing comprehensive evidence to inform clinical practice and policy interventions aimed at reducing neonatal deaths in the country.

## Methods

### Searches

A systematic literature search was conducted in PubMed, HINARI, Google Scholar, Embase, Scopus, and relevant Ethiopian university repositories to identify studies reporting the incidence and predictors of mortality among neonates with respiratory distress syndrome (RDS) in Ethiopia. In addition, a snowball search of reference lists from retrieved articles was performed to capture further relevant studies. To minimize publication bias, gray literature was systematically searched in the institutional repositories of Addis Ababa University, Bahir Dar University, Debre Berhan University, Hawassa University, University of Gondar, and Jimma University. Unpublished Master’s theses and dissertations reporting neonatal mortality outcomes were included if they met the predefined inclusion criteria.

The search covered studies published between January 1, 2000, and February 10, 2026, and was carried out from February 5 to 10, 2026. Both published and unpublished studies were included to provide a comprehensive estimate of the pooled incidence and its predictors.

A comprehensive search strategy was developed using keywords and Medical Subject Headings (MeSH) related to neonatal respiratory distress syndrome, mortality, and associated factors. The search terms included “incidence,” “survival status,” “survival,” “respiratory distress,” “respiratory distress syndrome,” “RDS,” “hyaline membrane disease,” “neonate,” “newborn,” “infant,” “baby,” “mortality,” “death,” “time to death,” “neonatal intensive care unit,” “NICU,” “factors,” “predictors,” “determinants,” “associated factors,” “risk factors,” “Ethiopia,” and “Ethiopian.” These terms were used both independently and in combination with the Boolean operators “OR” and “AND” to maximize search sensitivity (Supplementary File).

All retrieved records were exported to EndNote version 21.3 for management and duplicate removal. Remaining records were screened at title, abstract, and full-text levels. Studies were excluded if they were unrelated to the topic, conducted outside Ethiopia, or lacked sufficient data for analysis.

This systematic review and meta-analysis was conducted in accordance with the Preferred Reporting Items for Systematic Reviews and Meta Analyses PRISMA 2020 guidelines [14].

### Study inclusion and exclusion criteria

The inclusion criteria were defined using the PECOS framework:

#### Population

Studies conducted among neonates diagnosed with respiratory distress syndrome (RDS) in Ethiopia.

#### Exposure

A range of factors potentially influencing neonatal mortality in RDS were considered. However, factors were included in the analysis only if they were reported as associated with the outcome in two or more studies. These factors were low birth weight, prematurity, multiple pregnancy, perinatal asphyxia, and antenatal corticosteroid use.

#### Comparison

Not applicable.

#### Outcome

The primary outcome was the incidence of neonatal death among neonates with respiratory distress syndrome, occurring within 28 days of NICU admission and reported as deaths per 1000 neonatal-days. The study also assessed predictors of mortality.

#### Study design

All types of cohort studies, including prospective and retrospective designs, were eligible for inclusion. However, only retrospective cohort studies were included, as all relevant studies conducted in Ethiopia on this topic employed this design.

#### Time frame

Articles published between January 1, 2000, and February 10, 2026.

#### Country

Studies conducted exclusively in Ethiopia.

#### Language

Articles published in English.

#### Publication type

Both published articles and gray literature from university repositories were included to minimize publication bias.

### Exclusion criteria

After screening titles and abstracts, potentially relevant studies were assessed in full text. Studies were excluded if they did not report the outcome of interest (time-to-event incidence of neonatal mortality) or failed to meet other predefined inclusion criteria.

### Potential effect modifiers and reasons for heterogeneity

Potential sources of heterogeneity were explored using subgroup analyses based on region, publication year, study setting, statistical modeling approach, and sample size. These factors were selected because they may influence neonatal outcomes due to variations in healthcare access, clinical practices, study design, and population characteristics across settings in Ethiopia.

### Study quality assessment

The Joanna Briggs Institute (JBI) critical appraisal checklist for cohort studies was used to assess the quality of each included article. Each study was scored as a percentage of the total possible score. A cutoff of 50% was used to determine eligibility for inclusion in the review [15]. Two reviewers (YWA and AMM) independently evaluated the studies. The checklist consists of eleven questions with four possible responses: yes, no, unclear, and not applicable. Discrepancies in quality assessment were resolved by calculating the mean score of all reviewers’ evaluations.

### Data extraction strategy

Four authors (DME, TKY, AMW, and BFS) independently extracted all relevant data from the included studies using a standardized data extraction format prepared in Microsoft Excel. Any disagreements between reviewers were resolved by a fifth author (ABH) to ensure consistency and accuracy.

### Operational definitions

#### Incidence of neonatal mortality

was calculated in the included studies by dividing the number of deaths due to respiratory distress syndrome by the total neonatal-days of follow-up for all neonates measured within 28 days of life following admission to the neonatal intensive care unit expressed per 1000 neonatal-days.

#### Respiratory Distress Syndrome (RDS)

A clinical diagnosis in neonates characterized by tachypnea (> 60 breaths/min), bradypnea (< 30 breaths/min), apnea, expiratory grunting, nasal flaring, and chest wall retraction, with or without cyanosis, typically occurring in preterm infants or those with hyaline membrane disease.

#### Multiple pregnancies

A pregnancy in which two or more fetuses are carried simultaneously, including twins, triplets, or higher-order multiples.

#### Low Birth Weight (LBW)

birth weight less than 2500 g.

#### Very Low Birth Weight (VLBW)

Birth weight between 1000 and 1499 g.

#### Prematurity (Preterm Birth)

Birth occurring before 37 completed weeks of gestation.

#### Perinatal asphyxia

Failure to establish adequate breathing at birth, often indicated by Apgar score < 7 at 5 min, associated with hypoxia and acidosis leading to multi-organ compromise in the neonatal period.

### Data analysis and presentation

From each included study, data were extracted using a standardized form, including author, study area and region, publication and study year, setting, design, sample size, number of deaths, total neonatal follow-up days, and study quality score. Information on key predictors of mortality was also collected, including low birth weight, very low birth weight, preterm birth, antenatal corticosteroid use, multiple pregnancy, and perinatal asphyxia.

Extracted data were entered into Microsoft Excel and analyzed using Stata version 17. The primary outcome was the pooled incidence of neonatal mortality, calculated from the number of deaths and total neonatal follow-up time and expressed per 1000 neonate-days. Hazard ratios reported in the original studies were pooled to estimate the overall effect of predictors on mortality.

Heterogeneity was assessed using Cochran’s Q test and quantified with the I² statistic, which measures the proportion of total variation across studies due to heterogeneity rather than chance [16]. The I² values were interpreted as follows: 0–40% may not be important, 30–60% may represent moderate heterogeneity, 50–90% may represent substantial heterogeneity, and 75–100% may indicate considerable heterogeneity [17]. Forest plots were also examined to visually assess heterogeneity. A p-value < 0.05 from the Q test was used to declare statistically significant heterogeneity [16].

The pooled incidence of neonatal mortality was estimated using random-effects (DerSimonian and Laird) models [18], and results were presented in forest plots with 95% confidence intervals. Associations between mortality among neonates with RDS and potential predictors were assessed using pooled hazard ratios (HRs), with significance determined by a p-value ≤ 0.05 and the corresponding confidence intervals.

Sensitivity analysis was conducted for the pooled incidence of mortality to evaluate the robustness of the findings, although it was not performed for predictors due to the small number of included studies.

### Ethics approval and consent to participate

This study was conducted in accordance with the ethical principles of the World Medical Association Declaration of Helsinki. As this research was a systematic review and meta-analysis based solely on previously published studies and publicly available data, it did not involve direct human participants, patient contact, or identifiable personal data. Therefore, formal ethical approval and informed consent were not required, in line with national research ethics guidelines for secondary data analysis. All included studies were reviewed to ensure that they had obtained appropriate ethical approval and participant consent from their respective Institutional Review Boards (IRBs).

## Results

### Review statistics

**Fig. 1:**
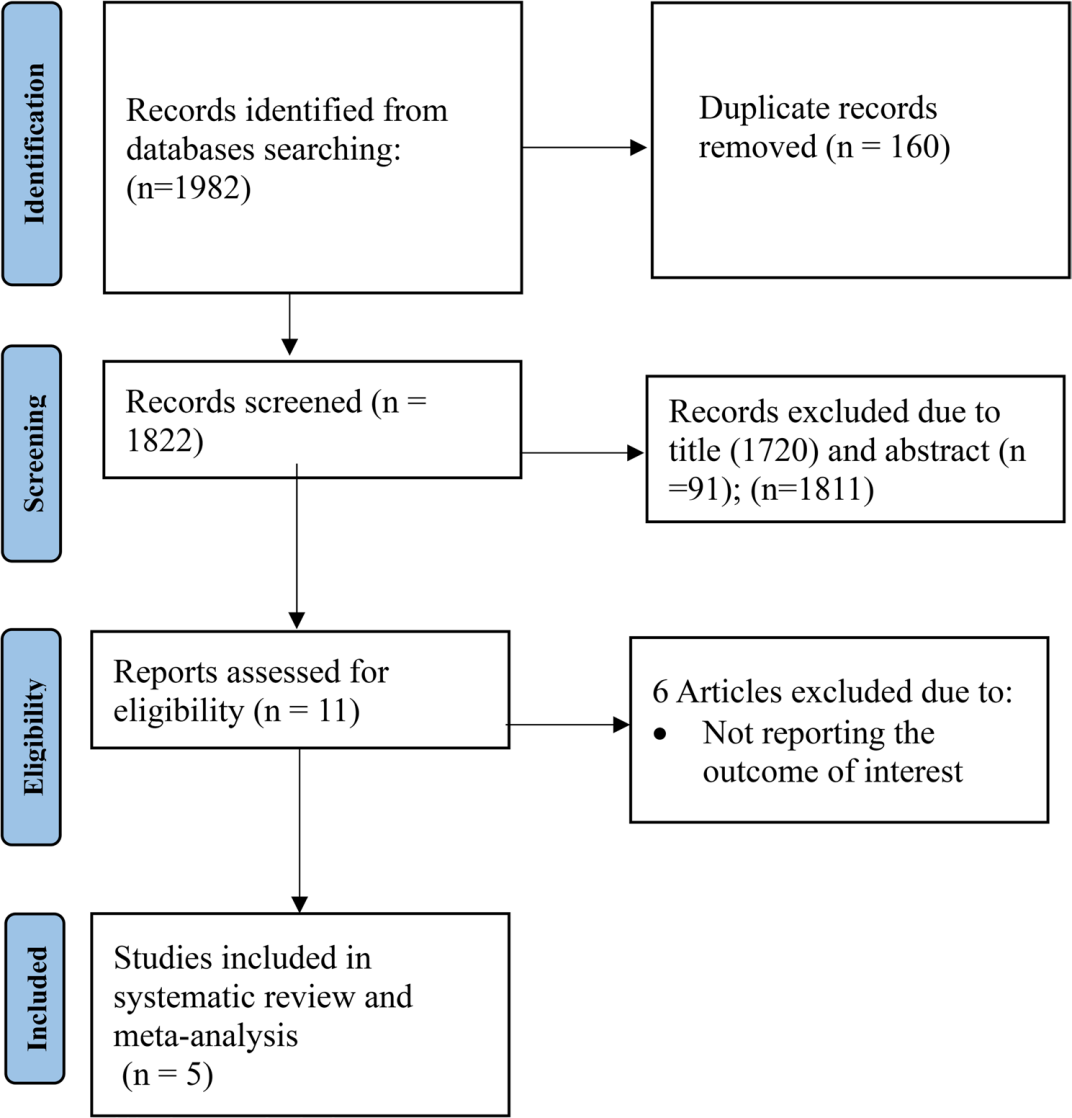
PRISMA flow diagram of included studies to estimate the incidence of neonatal mortality among neonates with respiratory distress syndrome in Ethiopia

A total of 1,982 published and unpublished studies were identified through electronic databases, including PubMed, HINARI, Google Scholar, Embase, Scopus, and digital library searches. After removing 160 duplicate records, the titles of the remaining articles were screened, resulting in the exclusion of 1,720 studies. An additional 91 articles were excluded after abstract review, and six full-text articles were excluded because they did not report the outcome of interest. Ultimately, five studies met the inclusion criteria and were included in the meta-analysis (Fig. 1).

A total of five retrospective cohort studies conducted between 2022 and 2025 were included in this systematic review and meta-analysis, comprising 2,121 neonates with respiratory distress syndrome admitted to neonatal intensive care units in Ethiopia. Three studies were conducted in the Amhara region, including single center studies from Debre Markos Comprehensive Specialized Hospital (DMCSH) [11] and Felege Hiwot Comprehensive Specialized Hospital (FHCSH) [19] and a multi-center study in East Amhara Comprehensive Specialized Hospitals [20], while two studies were conducted in West Oromia as multi center investigations [2, 13]. Regarding study settings, two studies were single center [11, 19] and three were multi center [2, 13]. All included studies used a retrospective cohort design. Concerning statistical approaches, two studies applied semi-parametric models [11, 20], while three used parametric survival models to identify predictors of mortality [2, 13, 19]. The sample sizes of the included studies ranged from 384 to 494 neonates. The number of deaths reported in individual studies ranged from 112 to 209. In total, the studies contributed 12,272 neonatal-days of follow-up. The largest sample was reported by Genet et al., 2023 [20] from East Amhara, while the smallest sample was from Gashaye et al., 2024 [11] at DMCSH (Table 1).

### Study quality assessment

All included studies scored above 70% on the Joanna Briggs Institute critical appraisal checklist for cohort studies, indicating moderate to high methodological quality. The highest score was reported by Bruck et al., 2023 (100%) [2], while the lowest score was 72.7% in Gashaye et al., 2024 [11]. Overall, the included studies demonstrated reliable data collection and outcome measurement, supporting the robustness of the pooled estimates (Table 1).

**Table 1:** Characteristics of included studies for the incidence and predictors of mortality among neonates with respiratory distress syndrome in Ethiopia

Authors and Year of Publication	Study Area	Region	Setting	Statistical Analysis	Design	Sample Size	Deaths	Neonatal-Days of Follow-up	Quality Score (%)	Source Type
Gashaye et al., 2024 [11]	DMCSH	Amhara	Single center	Semi-parametric	Retrospective cohort	384	141	1,864	72.7%	Peer-reviewed
Genet N., 2023 (Unpublished) [20]	East Amhara CSH	Amhara	Multi center	Semi-parametric	Retrospective cohort	494	209	3,472	81.8%	Gray literature
Bruck et al., 2023 [2]	West Oromia	Oromia	Multi center	Parametric	Retrospective cohort	406	152	2,539	100%	Peer-reviewed
Bruck et al., 2025 [13]	West Oromia	Oromia	Multi center	Parametric	Retrospective cohort	385	149	2,323	81.8%	Peer-reviewed
Selamsew K, 2022 (Unpublished) [19]	FHCSH	Amhara	Single center	Parametric	Retrospective cohort	452	112	2,074	77.3%	Gray literature

### Quantitative synthesis - meta analysis

#### Incidence of mortality among neonates with respiratory distress syndrome

**Fig. 2:**
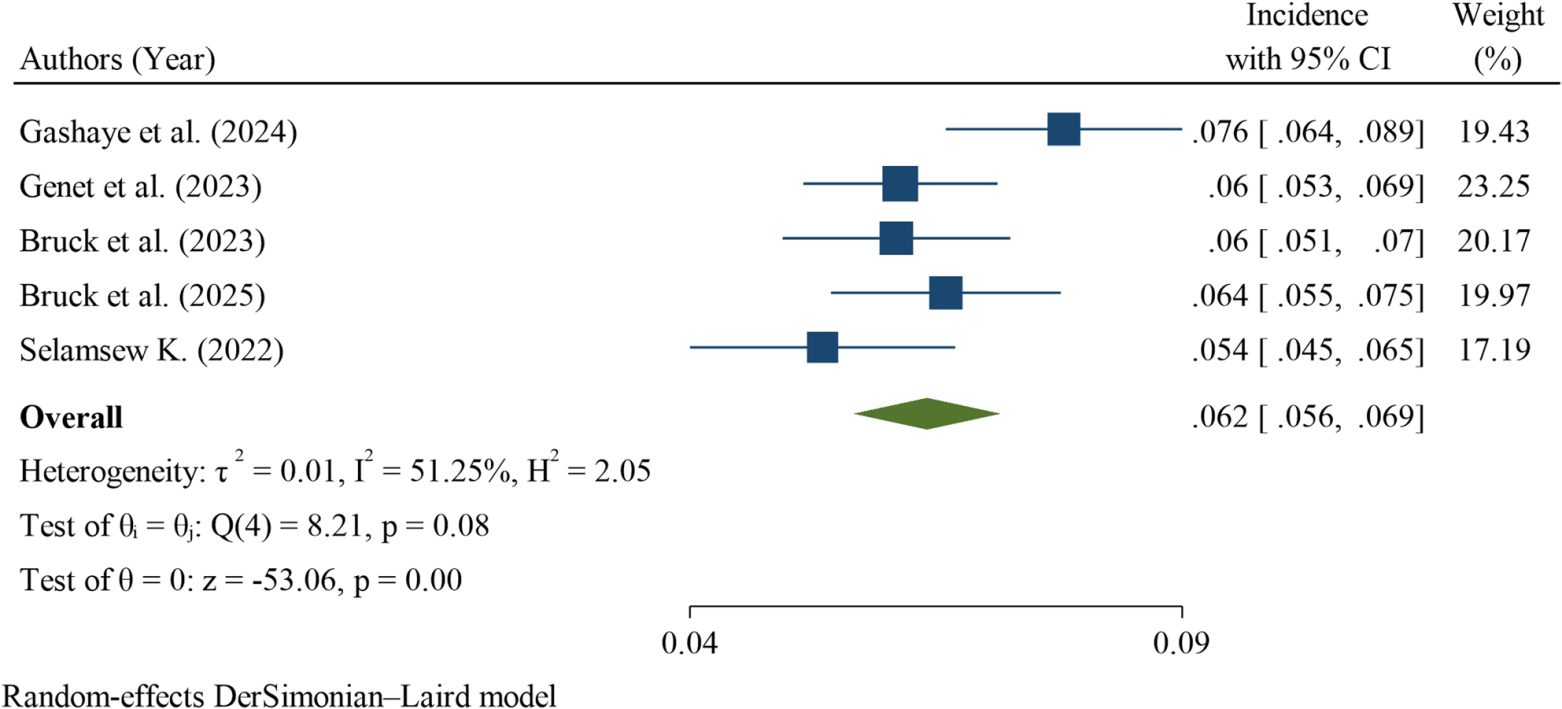
Forest plot of the incidence of neonatal mortality among neonates with respiratory distress syndrome in Ethiopia

Analysis of five studies showed that the pooled incidence of mortality among neonates with respiratory distress syndrome in Ethiopia was 62 deaths per 1,000 neonatal-days (95% CI: 56–69) across a total of 12,272 neonatal-days of follow-up, corresponding to a case fatality rate of 36% (95% CI: 32.5–40.1%) within 28 days of birth. Moderate to substantial heterogeneity was observed among the included studies (I² = 51.25%), and therefore the DerSimonian–Laird random-effects model was used to estimate the pooled incidence (Fig. 2).

### Heterogeneity test

**Fig. 3:**
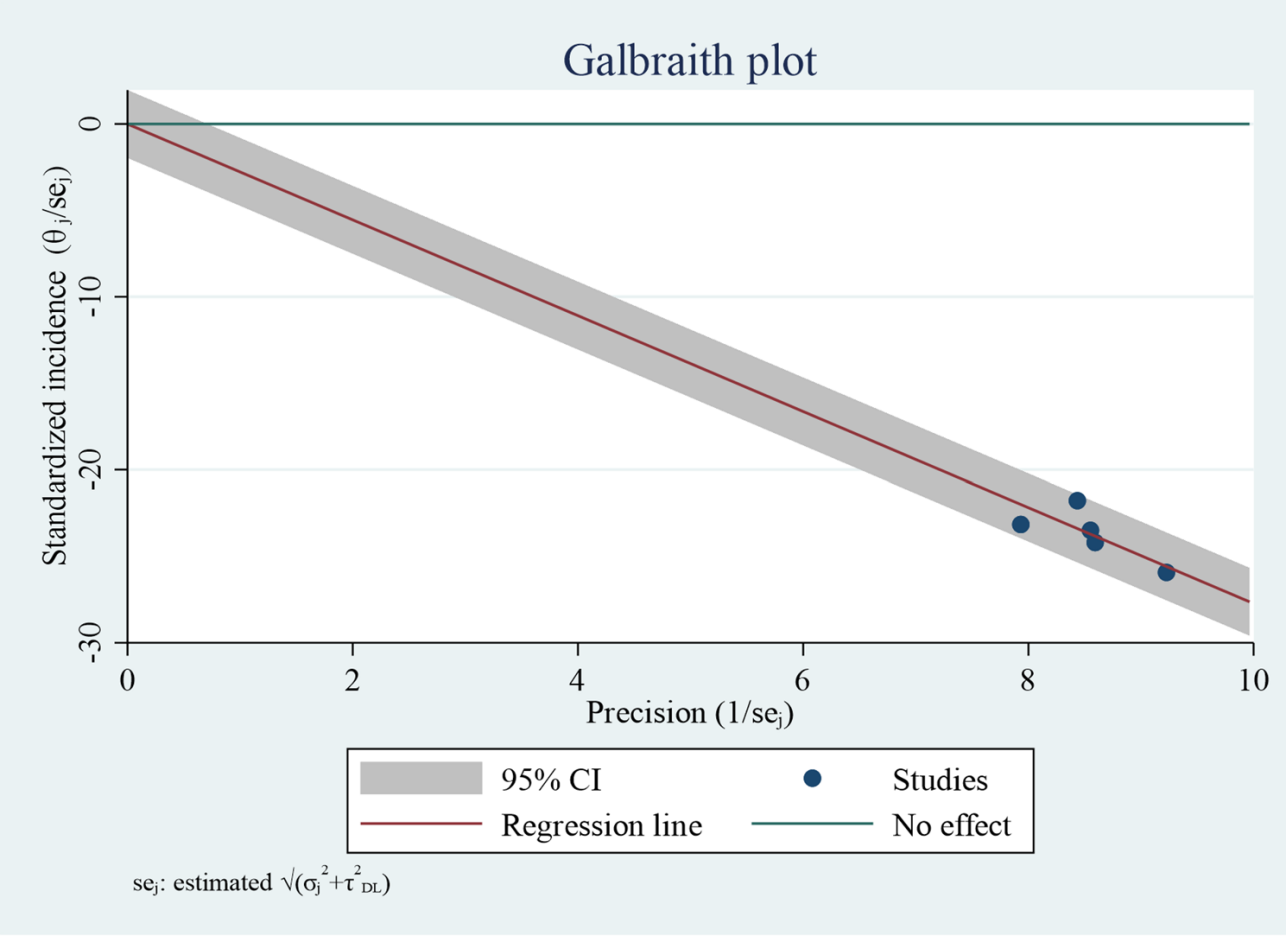
Galbraith plot testing heterogeneity of the incidence of neonatal mortality among neonates with respiratory distress syndrome in Ethiopia

The Galbraith plot showed that all included studies lay within the 95% confidence limits, indicating the absence of influential outliers. The plot suggested moderate heterogeneity, consistent with the I² statistic, but no single study disproportionately contributed to the observed heterogeneity (Fig. 3).

### Publication bias

**Fig. 4:**
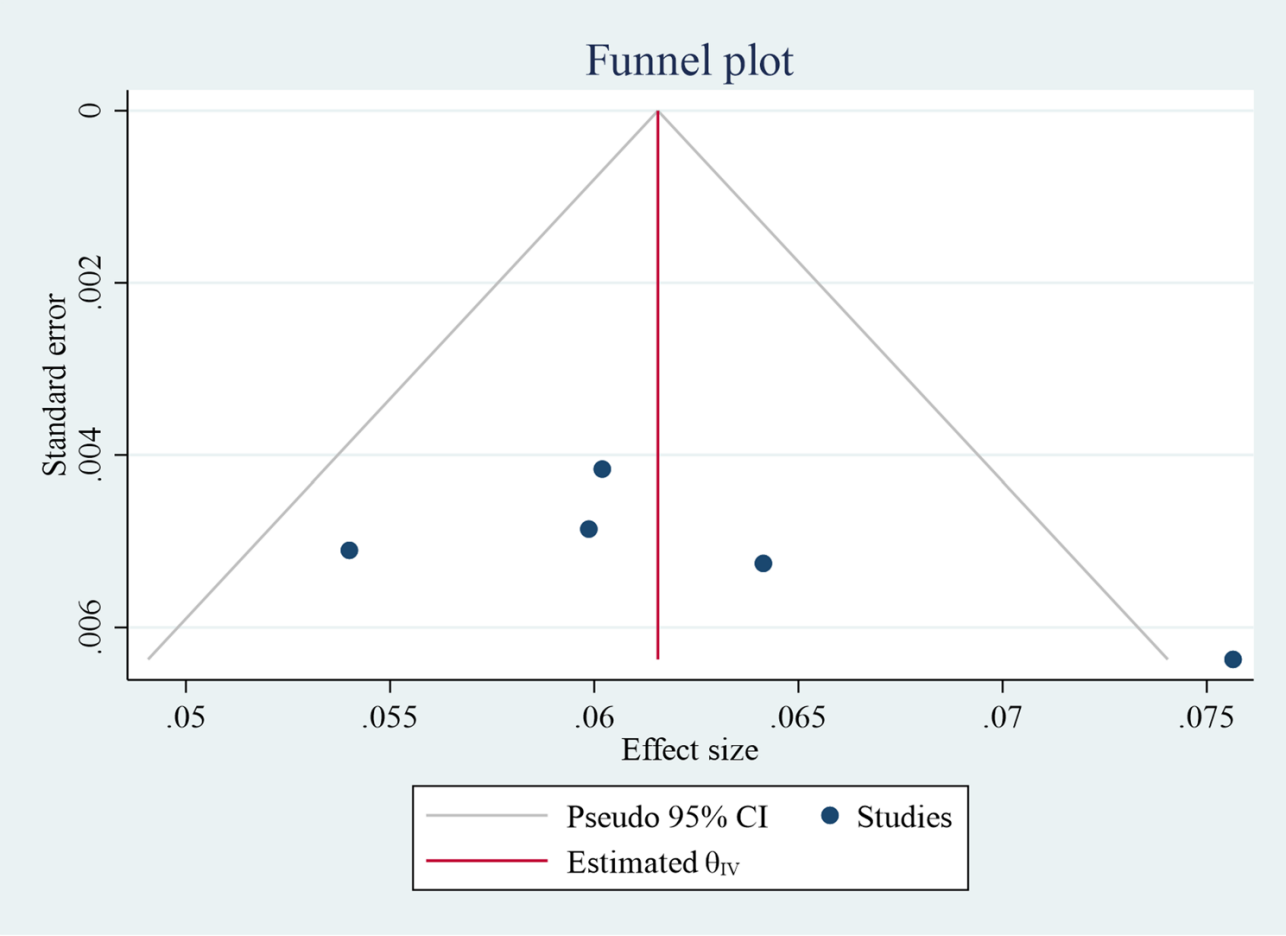
Funnel plot for inspecting publication bias for the incidence of neonatal mortality among neonates with respiratory distress syndrome in Ethiopia

Funnel plots and Egger’s tests, were used to assess publication bias [21, 22]. The funnel plot (Fig. 4) shows that the studies are roughly symmetrically distributed around the pooled effect estimate, with no evidence of substantial publication bias. This is supported by Egger’s test (t = 1.66, *p* = 0.196) (Table 2). However, given the small number of included studies (*n* = 5), interpretation should be made with caution.

**Table 2:** Egger’s test for testing publication bias for the incidence of neonatal mortality among neonates with respiratory distress syndrome in Ethiopia

Variable	Coefficient	Std. err	t	*P* > t	95% CI
Bias	6.54	3.94	1.66	0.196	-6.00, 19.08
Slope	0.029	0.02	1.46	0.241	-0.034, 0.093

### Sensitivity analysis

Sensitivity analysis (leave-one-out) indicated that the pooled estimate of respiratory distress syndrome mortality was robust and not unduly influenced by any single study. The effect size remained consistent at approximately 62 per 1000 when each study was removed individually, and the 95% confidence intervals were narrow and overlapped with the overall pooled estimate. Additionally, the consistently significant P values (e.g., *P* < 0.001) further supported the reliability of the findings (Table 3).

**Table 3:** Sensitivity analysis (leave-one-out) for the incidence of neonatal mortality among neonates with respiratory distress syndrome in Ethiopia

Omitted Study	Incidence per 1000 with 95% CI	*P*-value
Gashaye et al., 2024 [11]	60 (55–65)	< 0.001
Genet et al., 2023 [20]	63 (55– 72)	< 0.001
Bruck et al., 2023 [2]	63 (55– 72)	< 0.001
Bruck et al., 2025 [13]	62 (54– 71)	< 0.001
Selamsew K, 2022 [19]	64 (58– 71)	< 0.001

Random-effects DerSimonian-Laired model

### Subgroup analysis

The pooled incidence of mortality among neonates with respiratory distress syndrome across the five studies was 62 deaths per 1,000 neonatal-days (95% CI: 56–69), with an overall I² of 51.25%, indicating moderate to substantial heterogeneity. Subgroup analyses suggested that some categories, including parametric studies, multi-center studies, studies from Oromia, larger sample size studies, and studies published in 2023 or earlier, had little to no heterogeneity (I² = 0%), which might not be important. In contrast, semi-parametric studies (I² = 77.24%) and single-center studies (I² = 85.89%) showed substantial to considerable heterogeneity. Studies from the Amhara region (I² = 74.39%) also demonstrated substantial heterogeneity, while smaller studies with ≤ 450 participants (I² = 52.27%) had moderate to substantial heterogeneity. Studies published in 2024 or later exhibited moderate heterogeneity (I² = 49.27%). Overall, although subgroup analyses partially explained some of the variability, heterogeneity remained across certain categories, and differences between subgroups were not statistically significant (Table 4).

**Table 4:** Sub-group analysis for the incidence of neonatal mortality among neonates with respiratory distress syndrome in Ethiopia

Sub-group	Variables	Number of studies	Incidence per 1000 (95% CI)	Heterogeneity I²%	*P*
Statistical Analysis	Parametric	3	60 (54–66)	0.00%	0.39
	Semi-parametric	2	67 (54–84)	77.24%	0.04
	Overall	5	62 (56–69)	51.25%	0.34
Study Setting	Multi-center	3	61 (56–67)	0.00%	0.79
	Single-center	2	64 (46–87)	85.89%	0.01
	Overall	5	62 (56–69)	51.25%	0.79
Region	Amhara	3	63 (52–75)	74.39%	0.02
	Oromia	2	62 (55–69)	0.00%	0.55
	Overall	5	62 (56–69)	51.25%	0.90
Year of Publication	≤ 2023	3	59 (54–64)	0.00%	0.62
	≥ 2024	2	70 (59–82)	49.27%	0.16
	Overall	5	62 (56–69)	51.25%	0.07
Sample Size	≤ 450	3	66 (58–76)	52.27%	0.12
	> 450	2	58 (52–65)	0.00%	0.35
	Overall	5	62 (56–69)	51.25%	0.14

**Fig. 5:**
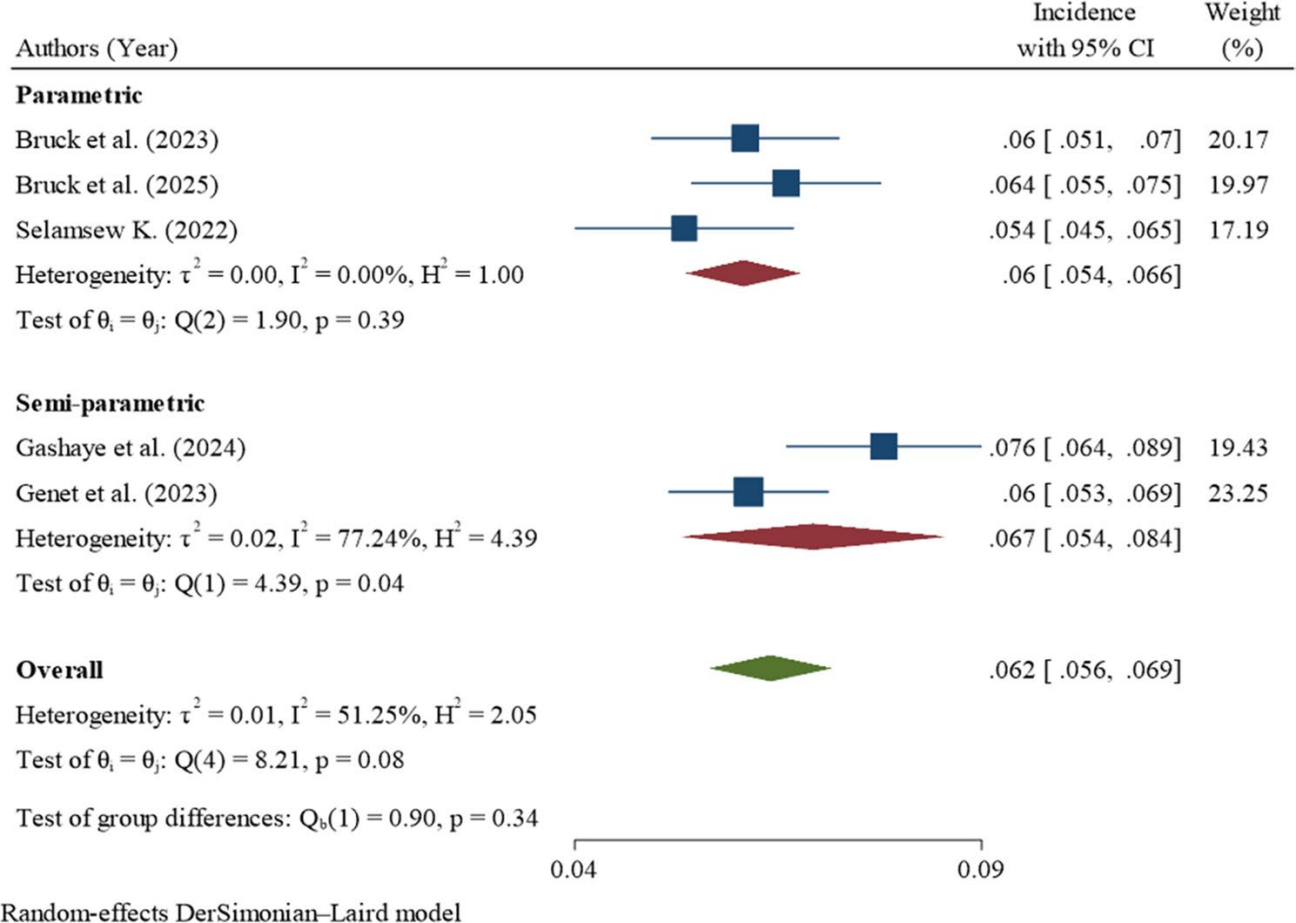
Sub-group analysis by type of analysis for the incidence of neonatal mortality among neonates with respiratory distress syndrome in Ethiopia

Semi-parametric studies showed substantial to considerable heterogeneity (I² = 77.24%). The elevated heterogeneity was primarily driven by two studies, Gashaye et al., 2024 [11] and Genet et al., 2023 [20] (Fig. 5).

**Fig. 6:**
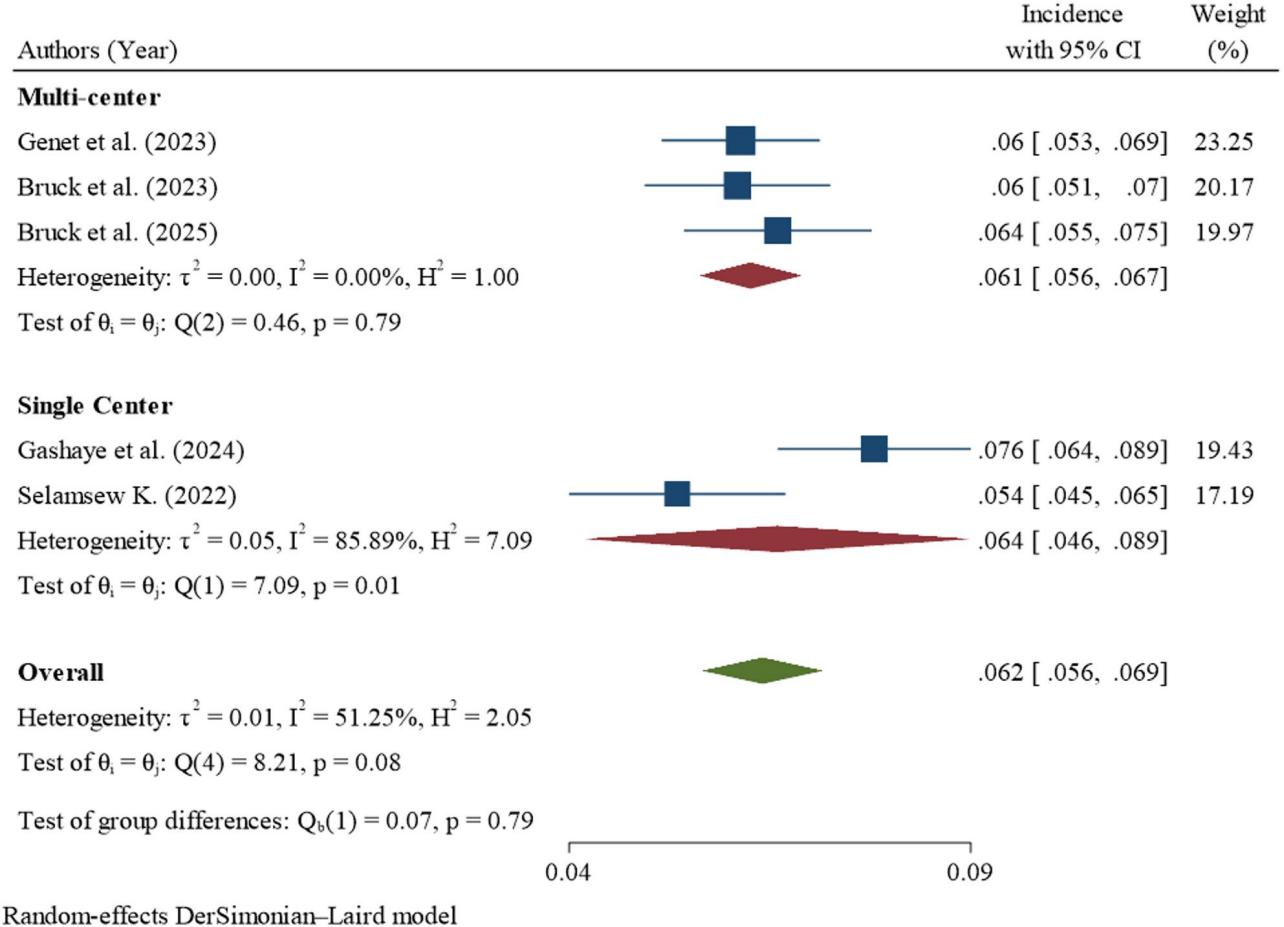
Sub-group analysis by study setting for the incidence of neonatal mortality among neonates with respiratory distress syndrome in Ethiopia

Single center studies showed substantial to considerable heterogeneity (I² = 85.89%). The elevated heterogeneity was primarily driven by two studies, Gashaye et al., 2024 [11] and Selamsew K, 2022 [19] (Fig. 6).

### Evidence of association

Several factors were assessed for their association with neonatal mortality related to respiratory distress syndrome. These included birthweight categories (1500–2499 g and very low birthweight 1000–1499 g), perinatal asphyxia, preterm birth, maternal corticosteroid use, and multiple pregnancies.

**Fig. 7:**
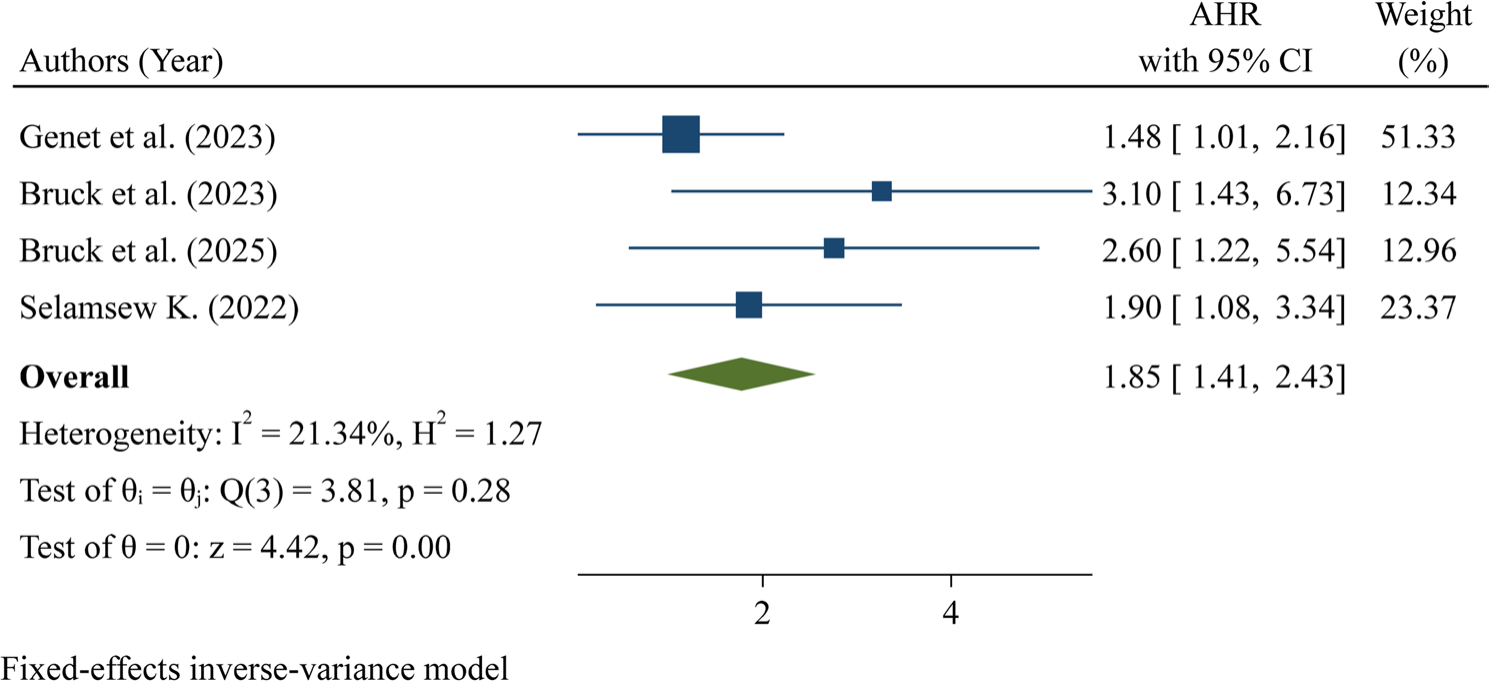
Pooled hazard ratio for birthweight 1500–2499 g as a factor associated with neonatal mortality among neonates with respiratory distress syndrome in Ethiopia

Birth weight between 1500 and 2499 g was analyzed in four studies and was found to be significantly associated with increased mortality among neonates with respiratory distress syndrome (RDS). The pooled hazard ratio (HR) was 1.85 (95% CI: 1.41–2.43), indicating that neonates within this birth weight range had an 85% higher risk of death compared with those of higher birth weight. Heterogeneity among the studies was low (I² = 21.34%; Q test *p* = 0.28), suggesting that the effect estimates were consistent across the included studies (Fig. 7).

**Fig. 8:**
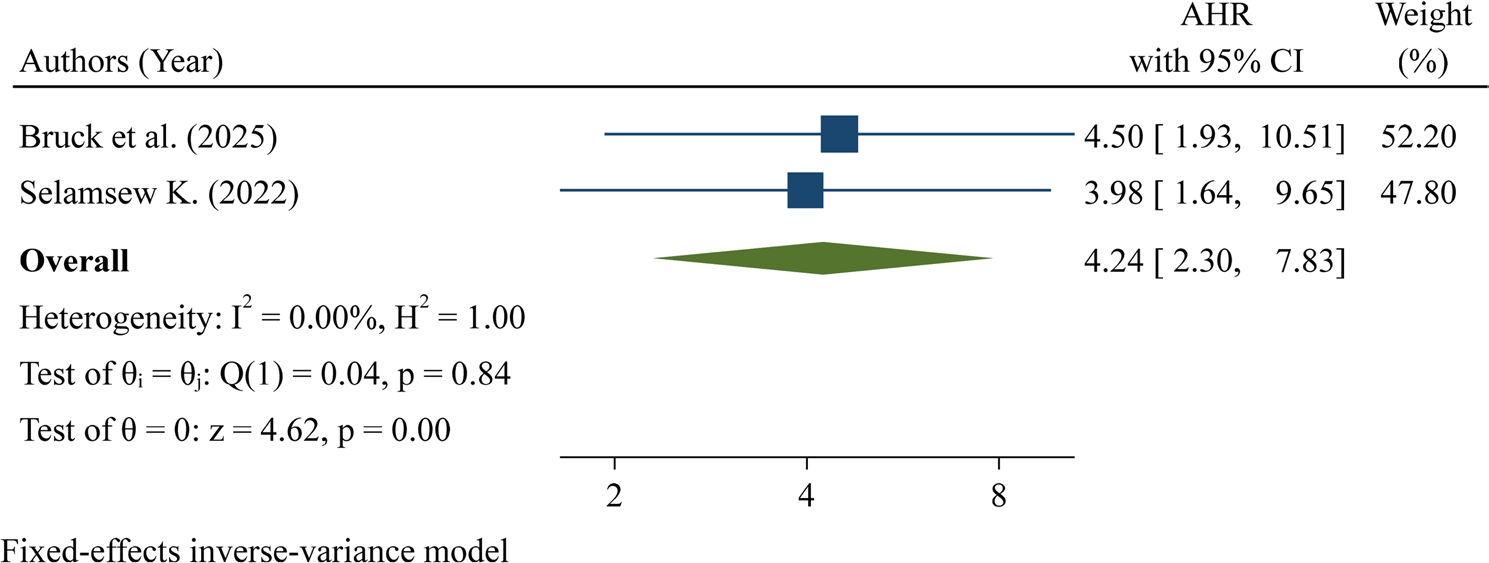
Pooled hazard ratio for birthweight 1000–1499 g as a factor associated with neonatal mortality among neonates with respiratory distress syndrome in Ethiopia

Very low birth weight neonates were analyzed in two studies. Both studies showed a significant association between very low birth weight and neonatal mortality. The pooled hazard ratio was 4.24 (95% CI: 2.30–7.83), indicating that neonates with very low birth weight had over four times the risk of death compared with neonates of higher birth weight. Heterogeneity was absent (I² = 0.0%; Q test *p* = 0.84), suggesting that the results were highly consistent across the two studies. The overall effect was statistically significant (z = 4.62, *p* < 0.001) (Fig. 8).

**Fig. 9:**
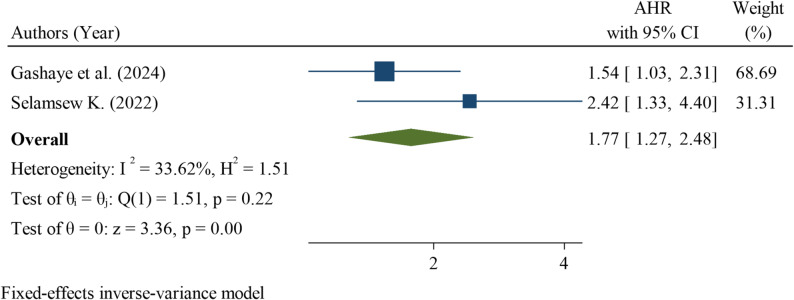
Pooled hazard ratio for preterm birth as a factor associated with neonatal mortality among neonates with respiratory distress syndrome in Ethiopia

Preterm delivery was assessed in two studies, both of which demonstrated a significant association with neonatal mortality among neonates with RDS. The pooled hazard ratio was 1.77 (95% CI: 1.27–2.48), indicating that preterm neonates had a 77% higher risk of death compared with those of higher gestational age. Heterogeneity was low (I² = 33.62%; Q test *p* = 0.22), suggesting that the findings were reasonably consistent across the studies. The overall effect was statistically significant (z = 3.36, *p* < 0.001) (Fig. 9).

**Fig. 10:**
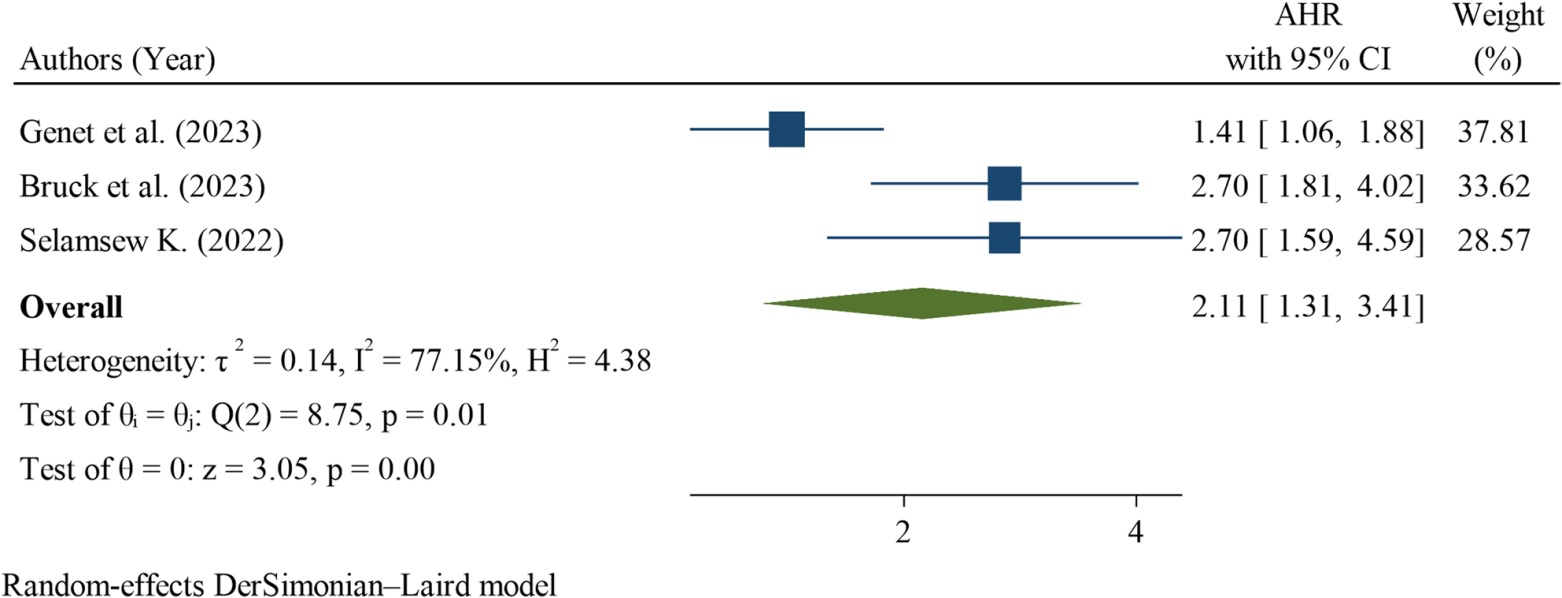
Pooled hazard ratio for perinatal asphyxia as a factor associated with neonatal mortality among neonates with respiratory distress syndrome in Ethiopia

Perinatal asphyxia was evaluated in three studies, all of which demonstrated a significant association with neonatal mortality among neonates with RDS. The pooled hazard ratio HR was 2.11 (95% CI: 1.31–3.41), indicating that neonates with asphyxia had more than twice the risk of death compared with those without asphyxia. Heterogeneity was high (I² = 77.15%; Q test *p* = 0.01), indicating substantial variability between the studies. The overall effect was statistically significant (z = 3.05, *p* < 0.001) (Fig. 10).

**Fig. 11:**
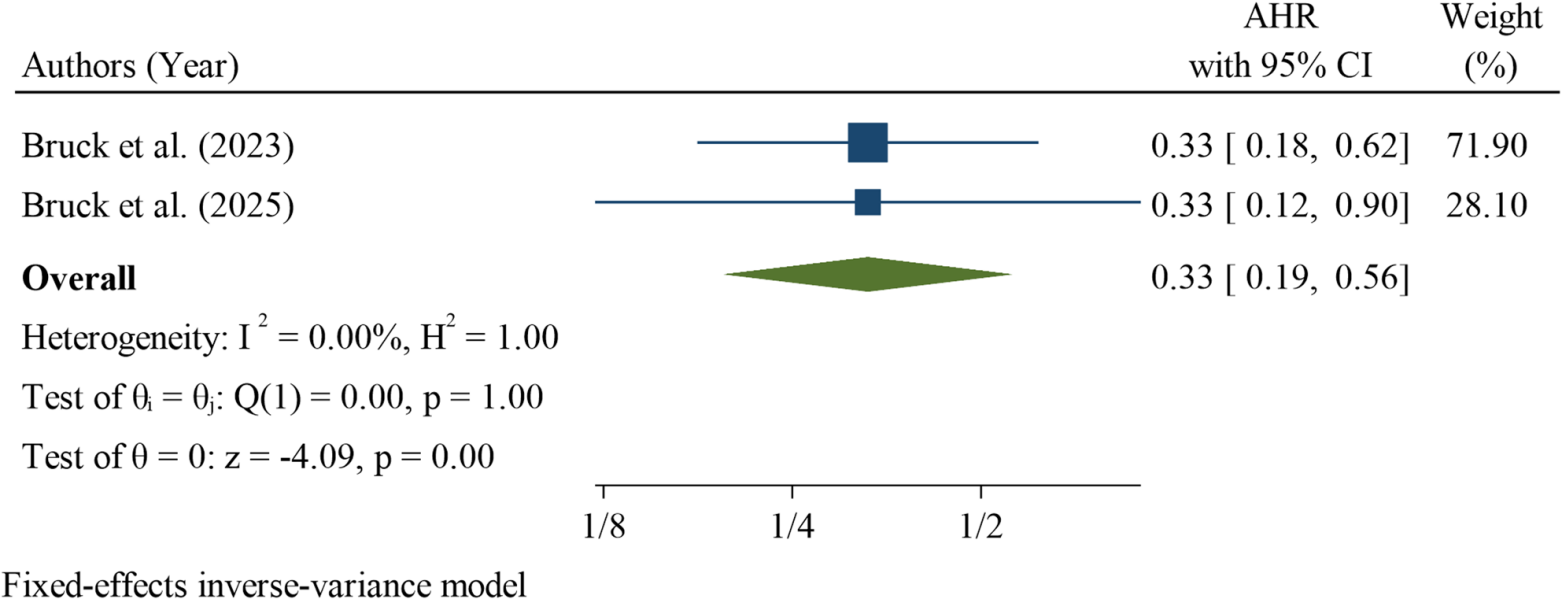
Pooled hazard ratio for maternal corticosteroid uses as a factor associated with neonatal mortality among neonates with respiratory distress syndrome in Ethiopia

Maternal corticosteroid use was evaluated in two studies, both of which demonstrated a significant association with reduced neonatal mortality among neonates with RDS. The pooled hazard ratio was 0.33 (95% CI: 0.09–0.56), indicating that neonates whose mothers received corticosteroids had a 67% lower risk of death compared with those whose mothers did not receive corticosteroids. Heterogeneity was low (I² = 0.00%; Q test *p* = 1.00), suggesting minimal variability between the studies. The overall effect was statistically significant (z = -4.09, *p* < 0.001) (Fig. 11).

**Fig. 12:**
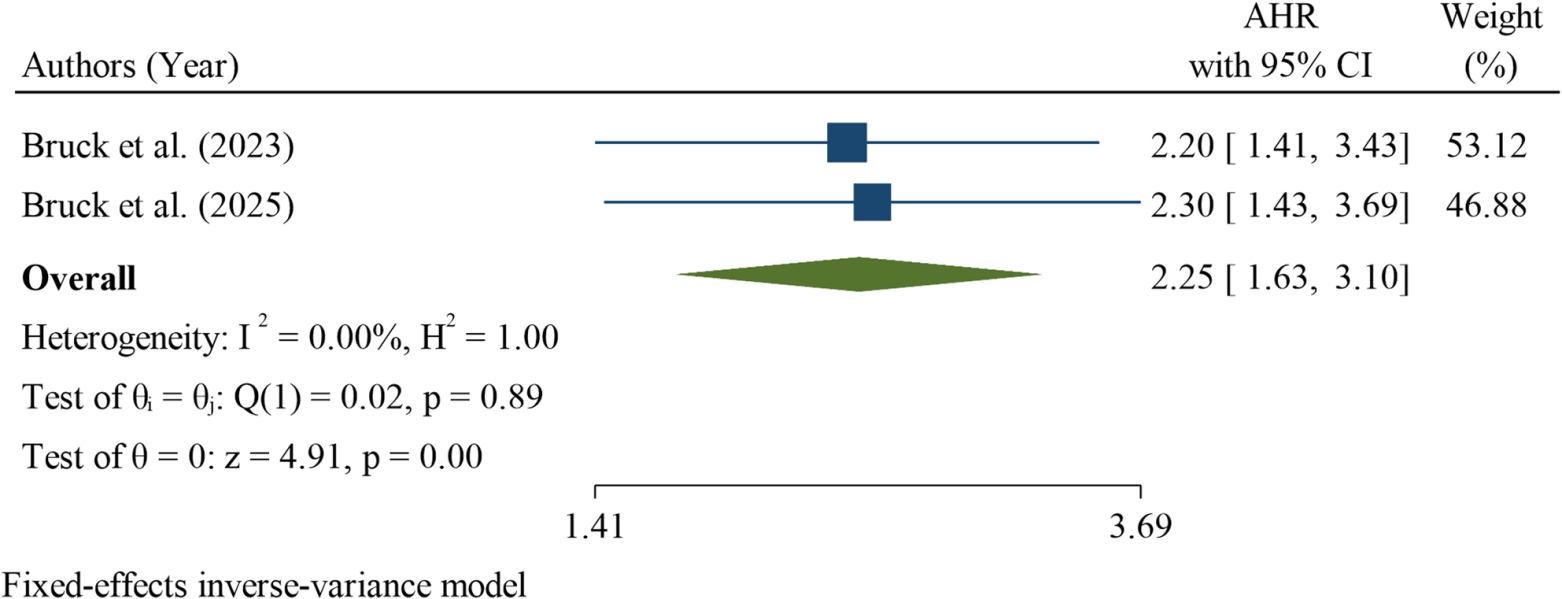
Pooled hazard ratio for multiple pregnancies as a factor associated with neonatal mortality among neonates with respiratory distress syndrome in Ethiopia

Multiple pregnancies (twins and higher-order) were evaluated in two studies, both of which demonstrated a significant association with increased neonatal mortality among neonates with RDS. The pooled hazard ratio HR was 2.25 (95% CI 1.63 to 3.10), indicating that neonates from multiple pregnancies had more than twice the risk of death compared with singleton neonates. Heterogeneity was low (I² = 0.00%; Q test *p* = 0.89), suggesting minimal variability between the studies. The overall effect was statistically significant (z = 4.91, *p* < 0.001) (Fig. 12).

## Discussion

This systematic review and meta-analysis estimated the pooled incidence and predictors of mortality among neonates with respiratory distress syndrome in Ethiopia based on evidence from two regional states. The analysis included five studies conducted only in the Amhara and Oromia regions due to the scarcity of available studies on this topic. Therefore, the findings may not be fully representative of the broader Ethiopian population. In emerging regions such as Somali, Afar, Benishangul Gumuz, and Gambella, where healthcare systems are relatively underdeveloped, limitations including fewer neonatal intensive care units, shortages of trained neonatal care providers, and restricted access to essential interventions such as continuous positive airway pressure and surfactant therapy may lead to higher incidence and mortality of respiratory distress syndrome. Furthermore, challenges related to geographic accessibility, weak referral systems, and delays in care seeking may further worsen neonatal outcomes in these settings [23].

Although only five studies were eligible for meta-analysis, a quantitative synthesis was considered appropriate because the studies were clinically and methodologically compatible with respect to population and outcome measures. Methodological authorities agree that there is no strict minimum number of studies required to perform a meta-analysis when the research question is clearly defined and the studies are sufficiently homogeneous with respect to their populations, interventions/exposures, and outcomes. The *Cochrane Handbook for Systematic Reviews of Interventions* explicitly states that while statistical tests for asymmetry such as funnel plots and Egger’s test are widely used to assess publication bias, these methods are *not recommended when the number of studies is fewer than ten* because they have low power to distinguish real asymmetry from chance and may lead to misleading conclusions [16]. Similarly, Egger and colleagues highlight that tests for funnel plot asymmetry are inadequate in small meta-analyses due to high rates of false positives and false negatives, reinforcing that such tests should be interpreted with extreme caution or avoided in small samples [21]. Instead, leave-one-out sensitivity and subgroup analyses were conducted to explore potential small-study effects and heterogeneity, and we interpreted the pooled estimates with caution, recognizing the limitations inherent in a small evidence base.

The analysis revealed a pooled neonatal mortality incidence of 62 per 1000 neonate-days (95% CI: 56–69) over a total of 12,272 neonate-days, corresponding to a case fatality rate of 36% (95% CI: 32.5–40.1%). The finding is consistent with a study in Bangladesh (36.5%) [24], and Tanzania (32%) [25]. This similarity may be explained by comparable country level health system contexts, as Ethiopia, Bangladesh and Tanzania are low-income settings with constrained neonatal care capacity [26, 27]. Limitations in access to advanced neonatal intensive care services, including surfactant therapy, mechanical ventilation, and continuous monitoring, may contribute to similar mortality outcomes [28]. In addition, these countries have a high burden of preterm birth and low birth weight, which are major risk factors for severe respiratory distress syndrome and increased mortality [29].

The finding is higher than a study in Nepal (15.96%) [30], India (14.5%) [31], Serbia (9.7%) [32], China (8.06%) [33], Saudi Arabia (5.1%) [34], and Nigeria (17.2%) [35]. This variation may be explained by differences in country level health system capacity and neonatal care infrastructure [26]. Countries such as China and Saudi Arabia have more advanced neonatal intensive care services, including wider availability of surfactant therapy, mechanical ventilation, and well-established neonatal monitoring systems, which significantly improve survival among neonates with respiratory distress syndrome [29]. Similarly, differences in early diagnosis, timely referral systems, and availability of skilled neonatal care providers may contribute to improved outcomes in these settings [36]. In contrast, resource limitations, higher burden of prematurity and low birth weight, and challenges in access to quality neonatal care in Ethiopia may contribute to the higher mortality observed [27, 37].

Moderate to substantial heterogeneity was observed among the included studies (I² = 51.25%). However, the substantial to considerable heterogeneity observed among single center studies (I² = 85.89%), compared to the absence of heterogeneity in multi center studies (I² = 0%), may be explained by variations in facility level characteristics. Single center studies are more likely to reflect the specific context of individual hospitals, where differences in neonatal intensive care unit capacity, availability of essential equipment such as continuous positive airway pressure and surfactant therapy, and variability in staff to patient ratios can significantly influence neonatal outcomes. Evidence indicates that neonatal outcomes vary considerably across centers due to differences in care practices, resource availability, and quality of services. In addition, variations in clinical management protocols, provider experience, and workload have been shown to contribute to differences in neonatal outcomes, and such variability may persist even after adjusting for patient level characteristics. In contrast, multi center studies pool data from multiple facilities, which can minimize the impact of local level variations and produce more consistent estimates with lower heterogeneity [38].

A birthweight of 1500–2499 g was associated with increased mortality among neonates with RDS, consistent with studies from Nepal [30], India [39], Macedonia [40], Serbia [20, 32], and Fiji [41]. Similarly, very low birth weight (1000–1499 g) significantly increased mortality risk, in line with evidence from India [9], Bangladesh [24], and Tanzania [25]. Lower birth weight reflects organ immaturity, particularly underdeveloped lungs with insufficient surfactant, which leads to poor gas exchange and severe respiratory compromise. Additionally, immature immune function and poor thermoregulation increase susceptibility to sepsis, hypothermia, and metabolic complications, further exacerbating the risk of death [2].

Antenatal corticosteroid use was associated with a lower risk of mortality among neonates with RDS, consistent with findings from Tanzania [25], India [39], and Fiji [41]. Corticosteroids accelerate fetal lung maturation and stimulate surfactant production, improving lung compliance and gas exchange after birth. This intervention also reduces the severity of RDS, decreases the need for mechanical ventilation, and lowers the risk of complications such as intraventricular hemorrhage and neonatal sepsis [42].

Multiple pregnancy was another predictor of higher mortality, consistent with evidence from São Paulo state, Brazil [43]. Neonates from multiple pregnancies, including twins and higher-order multiples, are more likely to be preterm and have low birth weight, which are key determinants of lung immaturity and inadequate surfactant production. Multiple gestations also increase the risk of intrauterine growth restriction, birth complications, and perinatal asphyxia, all of which compromise respiratory adaptation and heighten susceptibility to severe RDS [43].

Preterm birth was strongly associated with mortality among neonates with RDS, in agreement with studies from São Paulo state, Brazil [43], India [9], Macedonia [40], Serbia [32], and Fiji [41]. Preterm infants have underdeveloped lungs, insufficient surfactant production, and immature immune and thermoregulatory systems, which increase vulnerability to severe RDS, hypoxia, infection, and other life-threatening complications [44]. In the Ethiopian context, the management of preterm neonates is further challenged by limited availability of surfactant replacement therapy in government hospitals, the high cost of treatment, and insufficient continuous positive airway pressure (CPAP) machines along with difficulties in their maintenance. These resource constraints hinder effective treatment of preterm neonates and contribute to the persistence of high RDS-related mortality [45].

Finally, perinatal asphyxia was linked to higher mortality risk, consistent with evidence from Serbia [32] and Tanzania [25]. Hypoxia and acidosis at birth exacerbate pulmonary dysfunction, impair surfactant production, and compromise multiple organ systems. Neonates with RDS who also experience asphyxia are at increased risk of severe hypoxemia, metabolic derangements, and multi-organ failure, markedly elevating the likelihood of death compared with RDS alone [2].

### Strength and limitations

#### Strengths

This is the first systematic review and meta-analysis in Ethiopia to pool the incidence and predictors of mortality among neonates with respiratory distress syndrome, providing the first country-level quantitative evidence on this critical topic. A key strength of the study is the inclusion of unpublished data obtained from university repositories, which reduces publication bias and increases the comprehensiveness of the review. By pooling data from multiple studies, the analysis provides more precise estimates of incidence and hazard ratios for key predictors, offering actionable insights for clinical care and intervention planning. The study followed rigorous systematic review and meta-analysis methodology, including a clearly defined search strategy, eligibility criteria, and quality appraisal using the JBI checklist, ensuring transparency and methodological robustness. Sensitivity analysis for the pooled incidence further strengthens the credibility of the findings.

#### Limitations

Despite these strengths, the study has several limitations. First, only five studies from two regions of Ethiopia (Amhara and Oromia) were included, resulting in a small number of studies, reduced statistical power, and limited geographic representativeness. This scarcity of research may affect the precision and generalizability of the pooled estimates. Potential small-study effects cannot be excluded. Second, the included studies were primarily retrospective, introducing biases inherent to such designs, including selection bias, information bias from incomplete medical records, and variability in diagnostic or management practices. Several key confounders such as gestational age, birth weight, sex, birth asphyxia, sepsis, and respiratory support interventions (e.g., CPAP) were addressed in the individual studies, but residual confounding remains possible because other relevant variables, including antenatal corticosteroid use, timing of interventions, and quality of neonatal care, were not consistently recorded or adjusted for. Third, sensitivity analysis for predictors could not be performed due to the small number of included studies. For instance, the stability of hazard ratios for factors such as perinatal asphyxia, which showed high heterogeneity (I² = 77.15%), may have been influenced by the effect of individual studies. Finally, all included studies were published in English, as these were the only studies available. This may limit the inclusion of evidence from studies published in other languages, but reflects the availability of data.

## Conclusions

Neonatal mortality among neonates with respiratory distress syndrome in Ethiopia, based on evidence from two regional states, is high, with a pooled incidence of 62 per 1000 neonate-days and a case fatality rate of 36%. Key predictors of increased mortality include birth weights of 1500–2499 g and 1000–1499 g, preterm birth, multiple pregnancy, and perinatal asphyxia, while antenatal corticosteroid use significantly reduces the risk of death. These findings highlight the urgent need for targeted interventions, including promotion of antenatal corticosteroid administration, careful monitoring of high-risk neonates, and strengthening of neonatal intensive care services to improve survival outcomes in low-resource settings. Although these results provide important insights for clinical care and resource allocation, they are based on only five studies and should therefore be interpreted with caution. Future research from multiple regions of the country is recommended to enable a more robust meta-analysis and provide generalizable evidence across Ethiopia.

## Supplementary Information

Supplementary Material 1.

Supplementary Material 2.

## Data Availability

All data generated or analyzed during this study are included in this published article and its supplementary information files.

## Abbreviations

AHR: Adjusted Hazard Ratio
CI: Confidence Interval
DMCSH: Debre Markos Comprehensive Specialized Hospital
HR: Hazard Ratio
JBI: Joanna Briggs Institute
LBW: Low Birth Weight
NICU: Neonatal Intensive Care Unit
PNA: Perinatal Asphyxia
PRISMA: Preferred Reporting Items for Systematic Reviews and Meta-Analysis
RDS: Respiratory Distress Syndrome
VLBW: Very Low Birth Weight
