# Supplementary material for: Incidence and predictors of mortality among neonates with respiratory distress syndrome admitted to neonatal intensive care units in Ethiopia: a systematic review and meta-analysis of evidence from two regional states
Source: BMC Pediatr. 2026 Apr 17;26:502. doi: 10.1186/s12887-026-06877-5 (PMC13217728; doi:10.1186/s12887-026-06877-5)
Supplement: Supplementary file 1 — Supplementary Material 1. [file 12887_2026_6877_MOESM1_ESM.docx]

1. **PubMed Search Strategy**

(("Infant, Newborn"[Mesh] OR neonate*[tiab] OR newborn*[tiab] OR infant*[tiab] OR baby[tiab]) AND ("Respiratory Distress Syndrome, Newborn"[Mesh] OR "respiratory distress syndrome"[tiab] OR RDS[tiab] OR "hyaline membrane disease"[tiab] OR "respiratory distress"[tiab]) AND ("Mortality"[Mesh] OR "Infant Mortality"[Mesh] OR mortality[tiab] OR death[tiab] OR "neonatal mortality"[tiab] OR "incidence"[tiab] OR "survival status"[tiab] OR "time to death"[tiab] OR survival[tiab]) AND ("Ethiopia"[Mesh] OR Ethiopia[tiab] OR Ethiopian[tiab]) AND ("Risk Factors"[Mesh] OR factor*[tiab] OR predictor*[tiab] OR determinant*[tiab] OR "risk factor*"[tiab] OR "associated factor*"[tiab])) NOT ("systematic review"[pt] OR "meta-analysis"[pt]) AND Humans[Mesh] AND English[la] AND ("2000/01/01"[Date - Publication] : "2026/02/10"[Date - Publication])

1. **EMBASE Search Strategy**

(('newborn'/exp OR 'infant'/exp OR neonate*:ti,ab OR newborn*:ti,ab OR infant*:ti,ab OR baby:ti,ab) AND ('respiratory distress syndrome'/exp OR 'hyaline membrane disease':ti,ab OR 'respiratory distress syndrome':ti,ab OR 'respiratory distress':ti,ab OR RDS:ti,ab) AND ('mortality'/exp OR 'infant mortality'/exp OR mortality:ti,ab OR death:ti,ab OR 'neonatal mortality':ti,ab OR incidence:ti,ab OR 'survival status':ti,ab OR 'time to death':ti,ab OR survival:ti,ab) AND ('ethiopia'/exp OR ethiopia:ti,ab OR ethiopian:ti,ab) AND ('risk factor'/exp OR factor*:ti,ab OR predictor*:ti,ab OR determinant*:ti,ab OR 'risk factor*':ti,ab OR 'associated factor*':ti,ab)) NOT ('systematic review'/exp OR 'meta-analysis'/exp)AND [english]/lim AND [humans]/lim AND [2000-2026]/py

1. **HINARI Search Strategy:**

(("respiratory distress" OR "respiratory distress syndrome" OR RDS OR "hyaline membrane disease") AND (neonate OR newborn OR infant OR baby) AND (mortality OR "neonatal mortality" OR "neonatal death" OR death OR incidence OR survival OR "survival status" OR "time to death") AND (Ethiopia OR Ethiopian OR "East Africa") AND (factor* OR predictor* OR determinant* OR "risk factor*" OR "associated factor*")) NOT ("systematic review" OR "meta-analysis") AND English AND humans AND (2000:2026)

1. **Scopus** **Search Strategy**

(TITLE-ABS-KEY(neonate* OR newborn* OR infant* OR baby) AND TITLE-ABS-KEY("respiratory distress syndrome" OR RDS OR "hyaline membrane disease" OR "respiratory distress") AND TITLE-ABS-KEY(mortality OR "neonatal mortality" OR death OR incidence OR survival OR "survival status" OR "time to death") AND TITLE-ABS-KEY(Ethiopia OR Ethiopian OR "East Africa") AND TITLE-ABS-KEY(factor* OR predictor* OR determinant* OR "risk factor*" OR "associated factor*")) AND NOT TITLE-ABS-KEY("systematic review" OR "meta-analysis") AND (LIMIT-TO(LANGUAGE, "English")) AND (LIMIT-TO(DOCTYPE, "ar")) AND (PUBYEAR > 1999 AND PUBYEAR < 2027)

1. **Google Scholar Search Strategy**

("neonate" OR "newborn" OR "infant" OR "baby") AND ("respiratory distress" OR "respiratory distress syndrome" OR "RDS" OR "hyaline membrane disease") AND ("mortality" OR "neonatal mortality" OR "neonatal death" OR "death" OR "incidence" OR "survival" OR "survival status" OR "time to death") AND ("Ethiopia" OR "Ethiopian" OR "East Africa") AND ("factor" OR "predictor" OR "determinant" OR "risk factor" OR "associated factor")
